# Reflections on the Collaborative Story Analysis Method to Understand Qualitative Perspectives of Indigenous Syringe Services Program Clients

**DOI:** 10.1016/j.ssmqr.2024.100469

**Published:** 2024-07-26

**Authors:** Alexandra K. Perron, Brenna Greenfield, Atasha Brown, Frank Johnson, Toni Napier, Jordan Stipek, Aanji’bide Community Action Board, Jennifer J. Mootz

**Affiliations:** a University of Minnesota Medical School, Duluth Campus, 1035 University Dr, Duluth, MN, 55812, USA; b Columbia University Department of Psychiatry, New York State Psychiatric Institute, 1051 Riverside Dr, NY, 10032, USA; c Tribal Partner, MN, USA

**Keywords:** Qualitative analysis, Indigenous, Collaborative Story Analysis, CBPR, Storytelling, Stories

## Abstract

Many scholars have cautioned that the use of Western research methods is problematic in studies with Indigenous communities given colonialist histories that have exploited Indigenous populations. One solution has been to utilize a Community-Based Participatory Research (CBPR) approach to enhance equity in research partnerships. Employing a CBPR approach, however, does not necessitate the inclusion of Indigenous Research Methods, an additional step that can further benefit studies with their explicit alignment with Indigenous worldviews and values. In a CBPR project aiming to understand Indigenous harm reduction clients’ perspectives of barriers and facilitators to opioid use disorder treatment, our research group assembled a multidisciplinary qualitative data analysis team that included diverse tribal community members and academics. Sparse literature was available to guide the use of Indigenous Research Methods for the qualitative data analysis phase of the research. To address this gap, the aims of this process paper are: (1) to describe the implementation of the Collaborative Story Analysis method, and (2) in the Indigenous tradition of honoring and sharing stories, describe our analysis team’s experiences and perceptions of implementing this Indigenous Research Method. Through a series of process discussions, the analysis team found that applying the Collaborative Story Analysis method: (1) honored relationships and story, (2) strengthened the depth of analysis, and (3) exhibited tensions when working in a dominant Western culture. Through sharing our team’s experiences, the aspiration is that others can use these insights in their own consideration and implementation of an Indigenous Research Method for qualitative data analysis.

## 1. Introduction

Tribal communities in Minnesota continue to address the opioid crisis with innovative approaches as their communities are disproportionately impacted by opioids. In 2021, American Indians in Minnesota had 10 times the opioid overdose death rate compared to their white counterparts – higher than any other group in the state (Minnesota Department of Health, 2024). These inequities arise from and continue to be perpetuated by structural racism, exclusion from resources, and past and ongoing harm from settler institutions and governments.

Funded by a grant from the National Institute on Drug Abuse (R61/R33 DA049386), Aanji’bide (Changing our Paths) is a tribal and university partnership with a principal focus on learning from individuals using opioids to create new ways to advance culturally sensitive care. This study follows a Community Based Participatory Research (CBPR) approach – a model in which researchers partner with community members of an affected group. The intention is that this partnership will guide actionable change that benefits the community in an appropriate, welcomed, and effective manner (Krishnan et al., 2020). It is critical in a CBPR approach to involve community collaborators at every step of the research process – from crafting the research question, designing the methods, discussing and distributing results, and determining how these results should be translated into programs and policies (Krishnan et al., 2020). Principles of CBPR in tribal communities include identifying and recruiting community stakeholders to form a community advisory board, hiring from within the tribal community, frequent consultation with community advisory board, incorporating tribal feedback into all aspects of the project, and allowing the pace of the research to be set by the Tribe (Thomas et al., 2009; Tuhiwai Smith, 1999). Tribal Participatory Research (TPR) follows many of the same principles as CBPR, emphasizing collaborative efforts with community input at each step of the research process. TPR differs from CBPR in that there is a central focus on the unique historical and political relationships between tribal nations and the United States government, and between tribal communities and researchers and academic institutions (Beans et al., 2019). TPR focuses on the unique dynamics affecting tribal communities, including historical trauma, systemic racism, and the power dynamic between the United States government systems and tribal sovereign nations (Beans et al., 2019). However, neither CBPR nor TPR automatically include Indigenous *qualitative* research methods, a step that can provide a more decolonized analysis of data.

### 1.1. Research context

In a previous study, the Aanji’bide team conducted semi-structured interviews with Community Members who possessed expertise in opioid use disorder (OUD) treatment in a tribal setting in the Midwest. The study aimed to comprehend multi-level barriers and facilitators of OUD treatment. This includes how Indigenous cultural practices can be interwoven within OUD treatment, as well as identifying ways to enhance engagement at all stages of the OUD “Cascade of Care” (CoC; e.g., Medication Assisted Treatment (MAT) initiation and retention, relapse prevention, long-term recovery). The OUD CoC is a public health model that has been used to quantify different stages of engagement with OUD care at the population or systems level (Boyd et al., 2021; Piske et al., 2020; Socías et al., 2018; Williams et al., 2019; Yedinak et al., 2019). Interviewees were asked to comment on how the OUD CoC fit with an Anishinaabe worldview and the local Tribal Behavioral Health system (Boyd et al., 2021). Research participants highlighted that the OUD CoC model did not incorporate harm reduction services as a stage. Harm reduction includes a collection of strategies and ideas focused on reducing negative consequences associated with drug use (National Harm Reduction Coalition, 2020). The CoC also did not consider culturally relevant barriers and facilitators in tribal settings. Community Members perceived these as gaps (Boyd et al., 2021).

Thus, Aanji’bide facilitated a follow-up qualitative study within a tribally run Syringe Services Program to consider culturally relevant barriers and facilitators to accessing OUD treatment and MAT services in tribal settings. Goals of this work were to identify harm reduction clients’ perceptions of barriers and facilitators to accessing tribal OUD treatment programs; mental and physical health, including self-care; and their level of connection to culture and its relationship to opioid use.

Community partners helped design the methods. The Aanji’bide research collective includes a Community Action Board (CAB). The CAB consists of 11 Community Members who are active in addressing opioid use and behavioral health within the community. CAB members are tribally enrolled or descendants of the tribe. They have lived experience of opioid use, have family members who have used opioids, and/or work directly with individuals using opioids in the tribal healthcare system. The CAB met throughout the process to co-create the research study and ensure its relevance to community needs and inclusion of actionable parts. The CAB regularly attended meetings during the study and assisted with the development of the interview guide through several iterations (six drafts between June 2021–November 2021). They also provided commentary on results and paper development, reviewed the final paper, and provided their approval for data to be shared with a larger audience (nine dedicated CAB meetings September 2021–January 2024). Two of the 11 CAB members expressed additional interest in the coding, analysis and paper development and engaged in a more involved role on the qualitative data analysis team. Aanji’bide also collaborated with the tribe’s Syringe Services Program staff for input and further refinement (February 2023).

In March of 2022, the team conducted 22 interviews with 27 adult Indigenous clients who used non-prescription opioids within the last 30 days. Interviews were conducted at tribal Syringe Services Programs (five of the 22 interview sessions were completed in pairs). Slightly over half (52%) self-identified as female, while 48% self-identified as male. On average, participants were 39 years old with a range of 24–60 years old. In this geographically dispersed tribal nation, the Syringe Services Program rotates between several physical locations to allow for greater client accessibility. The program is staffed by public health nurses and provides access to sterile needles and equipment, health education, sexually transmitted infection testing, vaccinations, and naloxone. Other wraparound services, such as baseline medical assessments and connection to housing and treatment, are also available. Interviewees were harm reduction needle exchange clients who presented to the clinic for services. Recruitment was accomplished via approved flyers at the Syringe Services Programs and word of mouth by program staff. On the day of the interviews, program staff informed clients that the interviews were happening and where to go if they would like to learn more. The research team member provided information about the study and discussed eligibility criteria with interested participants. Eligibility criteria included: (1) active/established client at one of the tribal Syringe Services Programs; (2) 18 years or older; (3) identified as Indigenous; (4) used non-prescribed opioids within the last 30 days; and (5) had capacity and willingness to provide informed consent and participate. Research procedures were conducted in English. Following informed consent, the lead investigator administered a brief demographic survey and questionnaire about opioid use, and then conducted audio-recorded interviews. Clients could elect to participate individually or in pairs. We protected participant confidentiality by using an identification number to link demographic information with interview responses. No names or other discrete identifiable information were collected. Compensation for the interviewees consisted of a $20 prepaid Visa gift card and a small, culturally relevant gift (tea and sage salve). This study received approval from the University of Minnesota Institutional Review Board (IRB) and the Tribal IRB.

### 1.2. Qualitative analysis team

As few qualitative studies have included Indigenous community members in the data analysis process, we assembled a qualitative analysis team with the intention of including diverse voices and perspectives (Wendt & Gone, 2012). Including Indigenous community members can privilege Indigenous priorities, counteract researchers’ biases, and work against colonialist histories of subjugating research (Wendt & Gone, 2012). Our analysis team was made up of six members, with Indigenous (*n* = *4*) and non-Indigenous representation (*n* = *2*), CAB members (*n* = *3*), individuals in recovery from opioid use (*n* = *1*), university faculty (*n* = *1*), medical students (*n* = *2*), and graduate students *(n* = *1)*. Team members have backgrounds in tribal child protection, addiction counseling for a tribal nation, Nursing Director for a tribal nation, psychology, and holistic healing and herbal services for a tribal nation.

### 1.3. Beginning the search for an Indigenous qualitative analysis method

The original analysis plan was to use thematic analysis to code in pairs that contained at least one Indigenous coder and interpret the data using a codebook. To enhance the participatory process of data analysis, the team began by discussing perceptions of thematic analysis and collaboratively considered alternatives (Evans et al., 2009). The team, particularly the CAB members on the qualitative analysis team, perceived conventional qualitative analysis methods that break down narrative into discrete excerpts not to fit with Indigenous values that prioritize stories in a more holistic way. Traditionally, stories embody and honor relationships, fostering connections between the storyteller and receiver (Hallett et al., 2017). Additionally, using a thematic analytic method, each coding pair would closely read only about a quarter of the interviews. There was a consensus from the team members that a thematic approach would result in a loss of diverse voices. Following analysis discussions and conversations with the CAB, the team elected to explore other methods of qualitative data analysis based on Indigenous Research Methods. In essence, these methods arise from knowledge and traditions of Indigenous peoples and more closely represent the values of the participants and community partners. Indigenous Research Methods are grounded in relationships that are enhanced through storytelling. Such methods include an accountability to relationships with others, the land, and communities (Denzin et al., 2008; Windchief & San Pedro, 2019).

The analysis team began by conducting a literature search that specifically focused on describing Indigenous qualitative data analysis methods. However, this search unearthed few publications written on this topic and showed a significant gap in the literature. While finding Indigenous sources proved challenging, this gap does not stem from a lack of Indigenous wisdoms and ideologies. Indigenous knowledge is extensive and accessible, it is just not commonly found in the places where sources are found in Western academic research. Wendt and Gone (2012) reviewed studies that more broadly reported any qualitative research with American Indians. While they found 166 studies, they concluded that most of the qualitative studies used standard Western data analysis methods, such as Grounded Theory, phenomenology, case studies, narrative analysis, and hermeneutics. The authors provided several recommendations to address identified gaps: (1) provide sufficient detail about methods so other researchers can replicate or evaluate methods that take place with Indigenous communities, (2) write with clarity about approaches, and (3) avoid solely listing a data analysis approach (i.e., thematic analysis).

### 1.4. Paper objectives

In alignment with Wendt and Gone’s (2012) recommendations and acknowledgment of the literature gaps, the objectives of this process paper are to describe the way in which we selected and implemented the Indigenous Research Collaborative Story Analysis method. Additionally, as critical Indigenous pedagogy is characterized by reflexivity, decolonization, and transformation, we describe our qualitative analysis team’s experiences and perceptions (i.e., our story) of implementing this Indigenous Research Method. Our intent is that others can use these insights in their own consideration and implementation of an Indigenous Research Method for qualitative data analysis specifically. A future research article will include the findings of barriers and facilitators to opioid use treatment from the perspective of Syringe Services Program clients.

## 2. Process of selecting an Indigenous qualitative data analysis method

To learn about other researchers’ experiences in developing or adopting Indigenous qualitative data analysis methods, three qualitative analysis team members volunteered to present readings to the analysis team for consideration and discussion. The first article presented was “*Adapting Western Research Methods to Indigenous Ways of Knowing*” (Simonds & Christopher, 2013). In their case study with Montana State University and Crow Nation, qualitative analysis meetings that focused on themes or categories found within transcripts were perceived by community advisory board members as awkward and not fruitful. Members of their community advisory board preferred to share their own stories and experiences that related to the transcripts. Further, community advisory board members reminded the team that many Indigenous peoples learn through sharing stories and experiences. They elaborated that by breaking stories (interviews) up and dividing them into themes, the stories lose meaning and become difficult to relate to and understand. Community advisory board members also noted that in traditional ways, disassembling stories was disrespectful and dishonorable as the person telling it gives important context through their lived experiences. They concluded that an important part of a decolonized research method is the process itself – being flexible, adaptable, and listening (Simonds & Christopher, 2013).

The second article our team reviewed was “*Nanâtawihowin Âcimowina Kika-Môsahkinikêhk Papiskîci-Itascik*ê*win Astâcikowina [Medicine/Healing Stories Picked, Sorted, Stored]: Adapting the Collective Consensual Data Analytic Procedure (CCDAP) as an Indigenous Research Method*” (Starblanket et al., 2019). This paper discussed the formation of an adapted CCDAP technique for Indigenous, decolonized research. It offered a clear approach to adapting existing methods to make them more collective and Indigenous. The adapted CCDAP includes steps: (1) data collection, (2) data pre-analysis (broken down into minor themes/titles), (3) data digitization (minor themes presented as slides), (4) data reduction and display (table created with symbolized headers/titles, discuss where slides should be placed in table), and (5) conclusion and verification (group consensus and reflection, symbol replaced with original theme). These methods are centered around conversation to reflect, discuss, and organize qualitative data. The article highlighted the “Four R’s” in Indigenous ways – reciprocity, respect, relevance, responsibility. They noted that traditional ways are to share knowledge through storytelling and oral history. Thereby, the conversational component of a group discussion analysis method better aligns with Indigenous ways.

The third presentation described a book titled “*Research is Ceremony: Indigenous Research Methods*” (Wilson, 2008). This book shares that the foundation of research should be centered around respect, relationships, and reflection. Indigenous Research methods must be built on respectful relationships between researchers and between the researchers and the topic. It is important to reflect on each individual researcher’s role in the project, and responsibilities as a collective. Researchers must critically think about how these roles impact their relationship to the research topic and process. Finally, the team should reflect on how they are collectively and individually giving back in their roles and responsibilities and gauge the contribution to growth for all involved.

Finally, all team members read “*What Touched Your Heart? Collaborative Story Analysis Emerging from an Apsáalooke Cultural Context*,” a project that implemented a CBPR approach with a partnership among an Apsáalooke Community Advisory Board and Montana State University faculty and students (Hallett et al., 2017). Our team invited two of the paper’s authors, Suzanne Held of Montana State University and Alma Knows His Gun McCormick of Crow Nation, to speak virtually with our analysis team. They shared that elders and members of their community advisory board stressed the importance of preserving stories as a whole – a sentiment that our team felt was key when we initially started exploring other options for qualitative research that did not include open coding for excerpts and organizing excerpts into themes. They shared that their conversations seemed richer and more culturally aligned compared to using traditional Western qualitative analysis methods. They had meetings where food and stories were shared – a more traditionally Indigenous way to gather. Their team felt this method helped them to honor the storytellers, the content of their stories, and the sharing of mutual community.

## 3. Implementing the collaborative story analysis method

After considering the reference materials and reflecting on the guest speaker presentation, the team chose to analyze the data using a modified Collaborative Story Analysis, modeled after “What Touched Your Heart? Collaborative Story Analysis Emerging from an Apsáalooke Cultural Context” (Hallett et al., 2017). In the Hallett et al. (2017) analysis process, interviews were identified by a participant identification number or by participant name depending on preference and were introduced by the person who conducted the interviews. Each interview was prefaced with the following questions: “What really makes an impact on you? What touches your heart as you listen to this person’s story?” They then listened to the audio-recordings of interviews for approximately 30 min, followed by group discussion. All analysis team members (university and tribal Community Members) contributed to the conversations, with priority given to the Community Members. When the discussion had a natural close, they added two additional questions: “(a) ‘If you were to develop a program for this person to help them manage their CI [chronic illness], what would it look like?’ and (b) ‘How does this person’s story compare with others we have listened to?’” (Hallett et al., 2017). Of note, they had a total of twenty interviews to analyze.

Our team adopted the Collaborative Story Analysis Method with the understanding that part of using a decolonizing process is to be adaptable, flexible, and willing to shift when needed. Once the analysis method was selected, the team of 4–6 members met during a weekday evening virtually for 1.5 h weekly over a five-month period. Each transcript was shown to the team with the screen sharing feature on the virtual platform. In our process, the interviewer was not a part of the qualitative analysis team, so interviews were introduced by a participant identification number. For participant confidentiality, audio recordings were deleted after transcription and were not available for future listening. The team instead silently read the transcripts page-by-page. After each page was read, discussion ensued using the following guiding questions: “What stands out to you,” “What feels important,” and “What do you notice.” At the end of reading and discussing a transcript, team members reflected on the transcript as a whole and commented on any takeaway impressions about the interviewee’s narrative. The discussion leader each week, either university faculty or a medical student, would ask the discussion prompts and guide discussion. CAB members’ perspectives were prioritized through their sharing first. After CAB members shared their responses, discussion was opened for other team members’ input. Each session had a volunteer notetaker, usually a university faculty or medical student. Meeting notes included initials or names of team members’ comments and insights. We analyzed 22 interviews involving 27 participants.

## 4. Results of implementing collaborative story analysis

Throughout the analysis, the team held process discussions about experiences and perceptions of implementing Collaborative Story Analysis with a focus on what worked well, what could be improved or adapted, and how this work may guide future studies. When the analysis team completed conversations about all transcripts, the team held a retreat at a cabin near the tribal nation. This meeting began with sharing the study’s findings with Syringe Services Program personnel virtually and inviting them to comment on findings and provide input about next steps. The team then shared food and final reflections on experiences using the Collaborative Story Analysis method process. The first and last author reviewed process discussion notes and found that the team’s discussion points generally fell under three main topics. What follows is a description of the team’s perceptions of using Collaborative Story Analysis outlined according to these topics, which were: (1) benefits of honoring relationships and stories; (2) strengthened depth of analysis due to the collective conversations; and (3) an identified challenge regarding the tension between Western priorities and a decolonizing methodology that prioritized relationships. At times, quotations from team members are included as examples to illustrate perspectives.

### 4.1. Honoring of relationship and story

Overall, the method was well-received by the team. Research team members commented on how the process of conversation and discussion with each transcript prioritized honoring of relationships and story and that honoring was experienced throughout the process. One member shared, “Our culture is grounded in story. That’s how we learn. So it was nice to be able to look at people’s stories in depth.” Team members observed that two primary types of relationships were honored: relationship between the storyteller (interviewee) and the research team, and relationships amongst team members.

First, team members thought that the Collaborative Story Analysis method honored relationships with storytellers and the research team. They perceived the takeaway messages from participants to be holistic and meaningful when read together in their entirety. One research participant’s story, for example, included a comment that people should not be afraid to start treatment and should instead be encouraged. Team members then shared personal reflections with one another. For example, one team member shared, “People don’t need to be scared. You see people are court ordered. ICW [child protection] tells them they won’t see their kids. They’re forced to go, not encouraged.” Another team member noticed the shift in the interviewee’s attitude from the beginning of the interview to this comment at a later point, reflecting that “maybe the process of being asked these questions and having conversations changed something in this person about their perceptions.” Overall, team members thought that reading transcripts as a whole and as a group helped them feel closer to participants and understand them better.

Second, team members perceived the Collaborative Story Analysis method to create and strengthen relationships amongst the research team. Several members voiced they felt it brought the analysis team closer together. Analysis meetings included sharing of personal experiences, reactions, and emotions, offering a full embrace of team members’ positionality and subjectivity. Oftentimes, connections emerged between reactions and the context of members’ identities, and reflections occurred for how identities could be influencing interpretations of the data. Team members routinely brought unique understandings to interviewees’ comments based on differing life experiences. The group served as a safe space to be vulnerable together. Because the approach honored relationships in this way, team members shared that the process felt more lifegiving and meaningful than it might have to independently analyze data using a codebook.

Finally, one team member suggested, and others agreed, that one of the benefits of the Collaborative Story Analysis method is accessibility. This method does not require coding or research experience, and benefits from lived experiences. Collaborative Story Analysis allows individuals with different backgrounds, professional experiences, and worldviews to come together and form relationships through sharing stories. The accessibility of this method ensures a more equitable analysis wherein more voices and perspectives are represented and valued, particularly those of people from within the community being considered.

### 4.2. Strengthened depth of analysis

Team members thought the Collaborative Story Analysis method helped them capture the essence of what was being shared and identify intricacies within each story through use of a collective approach as compared to a more individualistic and traditional analysis method. Through analysis discussions, team members had the opportunity to share context based on expertise and knowledge of what services are available and stigma about topics within the tribal community. Throughout, the team commented on ways to improve future interviews and guides to elicit richer responses from participants. Team members appreciated learning about each other’s knowledge and context, bringing to light details that may have otherwise been overlooked.

For example, a topic that came up in nearly every participant’s story was transportation as a barrier to participating in OUD services. The analysis team had an extensive conversation surrounding this topic, even though many participants simply said “transportation” when they were asked about barriers to accessing treatment. One team member shared a story of a client who paid over $100 for a ride from Village #1 to Village #2 because the client did not have access to a car. Team members also shared anecdotes of geographical spread of different services: Village 1 has x service, but Village 2 has y service, which provided crucial context for team members located outside of communities and less familiar with available services on or near the tribal nation.

Another in-depth discussion happened about the story of a participant who had received a driving under the influence citation that resulted in prison time and the person losing their car and the ability to travel. The participant shared that extensive effort would be required to overcome losing their car and license. The analysis team discussed this person’s challenges and how they were rooted in a greater societal context. The team observed how oftentimes legal systems and policies meant to help and protect people can end up serving as additional barriers to accessing treatment through cascades of related outcomes. Team members talked about how when multiple systems are seemingly working against a person, it is understandable that the person may lose motivation or engagement to seek or continue treatment. The team furthermore reflected on the disconnect that is often present between authority figures and the effects of their power on people’s lives. For example, the law enforcement officer that removed the client’s car most likely did not consider how it may have resulted in the client losing their job, losing rights to their children, affecting finances, decreasing access to treatment, among other harmful outcomes.

The analysis team additionally reflected on the language and phrasing clients frequently used regarding treatment. For example, *passing* or *flunking* urinalysis and *failing* treatment were common descriptions used by participants. The team talked at length about the stigmatizing language of these words and how they could be seen as pathologizing and shame based. They reflected that these kinds of in-depth conversations about language may not have occurred had team members analyzed transcripts independently or in smaller groups of pairs.

The interview topics of mental health and self-care are additional examples of how the Collaborative Story Analysis method seemed to enhance the depth of the analysis process. Many participants simply responded “no” when asked the following question: “Many people who use opioids also live with physical and mental health struggles. Has this been part of your journey?” We also noted when asking participants the question about self-care, which was asked as a version of this question: “What do you do for self-care like physically? Mentally? Emotionally? Do you take time for a bath? A walk?” Participants often simply rephrased the example given by the interviewer or talked about meeting a basic need.

If the responses to these questions were coded as participants not experiencing mental health problems, then it could be inaccurately concluded that participants did not frequently experience mental health problems. However, the Collaborative Story Analysis method allowed for a rich discussion about mental health and how people were answering that specific question. Participants often used hesitant language when asked directly, leading the team to wonder about potential problems with how this question was phrased. The team thought about, and discussed the issue of stigma, and what mental health means to people. Additionally, the team reflected on how having an interviewer who did not live, work, or belong to the tribal nation ask these questions may have affected the interview responses. Team members shared many reflections on how questions were asked and received by interviewees. These conversations recognized phrases that may have been stigmatizing and troubleshooted ways to improve this in future studies. The team noticed that some of the interview guide questions may benefit from being reworded in future studies to phrase questions in a way that better connect with participants.

Perhaps most importantly, through collaborative discussions, the team observed that participants discussed grief and loss throughout the transcripts, and that those experiences were painful and difficult. Though the interviewer did not specifically ask about grief and loss, and most interviewees did not use that exact wording, many shared stories about losing loved ones and relatives and how those losses impacted their lives. Thus, experiences of grief and loss were interpreted as prevalent and potentially significant sources of mental health struggles, a topic that would benefit from further inquiry in future studies.

### 4.3. Potential tensions with collaborative story analysis

While the team generally favored the Collaborative Story Analysis method, they also identified some potential drawbacks to its use. There was an inherent tension between this decolonizing process and a Western cultural emphasis on productivity and task orientation.

Western and academic culture generally places high value on productivity, efficiency, and individuality. In contrast, the Collaborative Story Analysis method is a community-centered, rather than individually driven, method. By way of viewing stories as a whole and analyzing them collectively in a community team setting, the process is time intensive and thereby may be “less efficient” than popular qualitative analysis methods, such as thematic analysis or grounded theory methods. Analysis meetings required coordinating schedules of 4–7 people with different professional, academic, and life responsibilities. Meetings averaged 1.5 h each week, with the meetings being held in the evenings outside of conventional work hours. While this method was not overly burdensome with our relatively small number *(n* = *22*) of brief transcripts (interviews were between 15 and 20 min), the analysis process took several months to complete. Team members acknowledged that implementing a Collaborative Story Analysis method could become problematic for larger sample studies with longer interviews and transcripts. For those whose work is situated in Western academic institutions that largely define productivity in terms of product creation and dissemination, there can be tension between institutional expectations and implementing a method that requires more time. Despite the slower process with less structure, the team agreed that the drawbacks were worth the perceived gains of adopting this approach.

Finally, Collaborative Story Analysis does not produce standardized themes and requires harnessing intuition, emotion, and bringing the full self to the analysis process. Typically, researchers analyze qualitative results by identifying patterns and themes across interviews and selecting quotations from various interviewees that support those themes. While the team did synthesize some themes throughout the process, team members wrestled with whether this synthesis honored the holistic stories. There remains uncertainty about methods to pursue for final analysis iterations and ways to present the research findings that align with the collaborative method and process. Ideas for next steps are to present some overarching themes found across participants’ stories and use longer quotations to show context or story (e.g., a full interview) in its entirety to illustrate the themes. Alternatively, an Indigenous storyteller could create a story based on themes that cut across participants’ experiences. However, there were not many examples in the literature of decolonized, story-based qualitative analysis, which made interpretation of the results more challenging. Further work is needed in this area.

## 5. Conclusion

This article is noteworthy for overviewing the process of implementing the Indigenous Collaborative Story Analysis method (Hallett et al., 2017) and offering a detailed account of a diverse qualitative analysis team’s experiences and perceptions of using this method. The team observed that the Collaborative Story Analysis method offered a holistic approach to qualitative research, particularly in the context of Indigenous research. The method’s emphasis on story-sharing and honoring relationships kept Indigenous traditions and values centered throughout the entire process. The team also found that employing this method not only honored and strengthened relationships but also enhanced the analysis, providing valuable insights to guide future research studies. However, prioritizing relationships over productivity and efficiency could potentially create tensions with the expectations of Western institutions for outputs. This highlights the need for further exploration of Indigenous and collaborative research within qualitative research literature.
